# Highly reliable wind-rolling triboelectric nanogenerator operating in a wide wind speed range

**DOI:** 10.1038/srep33977

**Published:** 2016-09-22

**Authors:** Hyungseok Yong, Jihoon Chung, Dukhyun Choi, Daewoong Jung, Minhaeng Cho, Sangmin Lee

**Affiliations:** 1 School of Mechanical Engineering, Chung-Ang University, 84, Heukseuk-ro, Dongjack-gu, Seoul 156-756, Republic of Korea; 2 Department of Mechanical Engineering, College Engineering, Kyung Hee University, 1732, Deogyeong-daero, Giheung, Yongin, Gyeonggi 446-701, Republic of Korea; 3 Aircraft System Technology Group, Korea Institute of Industrial Technology (KITECH), Yeongcheon-si, Gyeongbuk-do, 38822 Republic of Korea

## Abstract

Triboelectric nanogenerators are aspiring energy harvesting methods that generate electricity from the triboelectric effect and electrostatic induction. This study demonstrates the harvesting of wind energy by a wind-rolling triboelectric nanogenerator (WR-TENG). The WR-TENG generates electricity from wind as a lightweight dielectric sphere rotates along the vortex whistle substrate. Increasing the kinetic energy of a dielectric converted from the wind energy is a key factor in fabricating an efficient WR-TENG. Computation fluid dynamics (CFD) analysis is introduced to estimate the precise movements of wind flow and to create a vortex flow by adjusting the parameters of the vortex whistle shape to optimize the design parameters to increase the kinetic energy conversion rate. WR-TENG can be utilized as both a self-powered wind velocity sensor and a wind energy harvester. A single unit of WR-TENG produces open-circuit voltage of 11.2 V and closed-circuit current of 1.86 μA. Additionally, findings reveal that the electrical power is enhanced through multiple electrode patterns in a single device and by increasing the number of dielectric spheres inside WR-TENG. The wind-rolling TENG is a novel approach for a sustainable wind-driven TENG that is sensitive and reliable to wind flows to harvest wasted wind energy in the near future.

The development of renewable energy sources is one of the most important challenges in today’s world due to the rapid increase in industries and fossil fuel consumption. As wind energy harvesting is a sustainable and clean power source, kinetic energy from windmills was used to generate electricity through wind turbines and to thereby harvest wind energy. Current methods to generate electricity include conventional wind turbines using electromagnetic generators. However, they have numerous drawbacks such as complicated fabrication, inconstant electricity production, and limited construction location^1^,^2^,^3^. Recently, several methods such as utilizing piezoelectric and triboelectric effects were proposed to overcome the difficulties of the wind turbines^4^,^5^,^6^,^7^,^8^,^9^,^10^,^11^,^12^,^13^,^14^,^15^,^16^. Among the wind energy harvesting methods, a triboelectric nanogenerator (TENG) is a very important generating mechanism due to its simple design, input sensitive output, and high power density. TENG converts mechanical movements such as rotation and reciprocation to electrical energy by the contact electrification between two triboelectric materials and the electrostatic induction effect. Various studies indicated that TENGs could be used as wind power generators and self-powered sensors by inducing rotation or vertical contact-separation motions using windmills^13^,^14^,^15^ and the fluttering behavior of flexible substrates^4^,^6^,^7^,^8^,^9^,^16^. However, the robustness and durability of the wind-driven TENGs continue to pose challenges due to the wear and fatigue failure caused by the friction between two dielectric materials and a repeatedly applied load during flutter motion. Furthermore, predicting the precise movement of wind flow is essential to achieve a wind flow sensitive TENG that can harvest even the slightest wind. Nevertheless, there were only a few attempts to force the movement of devices by an enclosed chamber or blades^17^ and to observe the movement of the fluttering material through a high-speed camera^6^. There are no studies yet demonstrating a dynamic analysis of the wind flow in conjunction with an optimized design of wind-driven TENGs. A new design and analysis for wind flow in the devices is necessary for sustainable and robust wind-driven TENGs with sensitive wind flow outputs.

This study demonstrated and optimized parameters for a wind-rolling triboelectric nanogenerator (WR-TENG) by using dynamic simulation to harvest a wide range of inputs. The vortex whistle design has an entrance and an exit that created a large vortex flow as air passed through the device^17^. In addition to the wind flow, a lightweight sphere-shaped dielectric moved along with the vortex flow and contacted the electrode on the inner surface of the whistle in the cases with strong winds and weak winds. In the study, computation fluid dynamics (CFD) simulations are used for the first time to optimize the flow inside the TENG and the movement to efficiently convert wind energy to kinetic energy. CFD can visualize the complex vortex flow and provide design parameters for improving the stability of the orbit of the lightweight sphere-shape dielectric. Moreover, electrodes inside the device are patterned to have multiple outputs in a single rotation to increase the space-efficiency of the WR-TENG. The self-powered anemometer based on the WR-TENG with multiple electrodes generated linear signals of electrical output ranging from a weak wind (2 m/s) to a strong wind (exceeding 30 m/s). Furthermore, the whistle-shaped wind energy harvester with free-standing mode TENGs and multiple sphere dielectrics could generate a maximum rectified open-circuit voltage (*V*_*OC*_) of 11.2 V and a short-circuit current (*I*_*SC*_) of 1.86 μA. This new approach is a potential solution for wind-driven TENG design, and for practical applications of a compact and lightweight wind power generator.

## Result and Discussion

**Figure 1:**
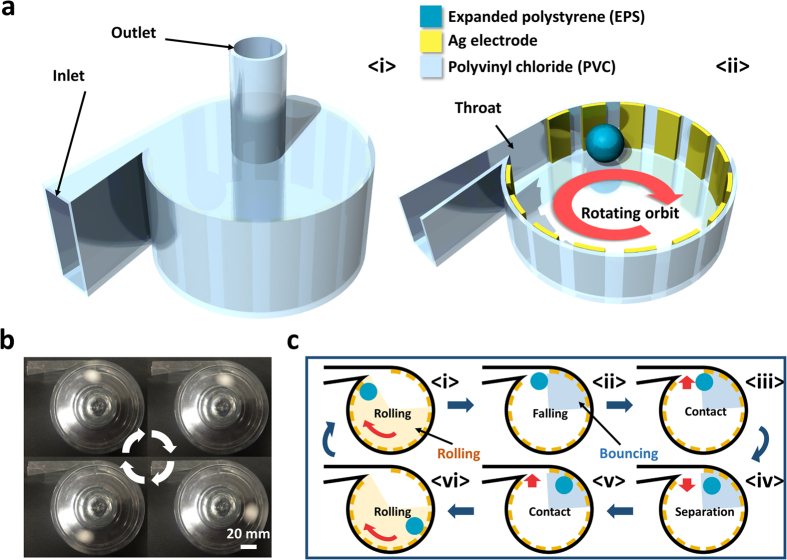
The wind rolling triboelectric nanogenerator. (**a**) The schematic illustration of the inner structure of the WR-TENG. (**b**) Photograph of the EPS sphere rotating inside the WR-TENG (air flow velocity of 2.2 m/s). (**c**) The working mechanism of a WR-TENG.

As shown as Fig. 1a, a wind-rolling triboelectric nanogenerator (WR-TENG) was based on a 100 mm diameter vortex whistle. An EPS sphere was used as a dielectric to easily obtain the kinetic energy from the wind energy to induce a rolling motion along the inner surface of the vortex whistle. The density of an EPS is approximately 115 times lower than that of fluoropolymer materials (EPS = 19.1 kg/m^3^, PTFE = 2200 kg/m^3^), which are among the common dielectrics used in TENGs, and this enables WR-TENGs to generate electricity even in the presence of a slight wind. The light-weight expanded polystyrene (EPS) has a low centripetal force and low friction for the WR-TENG to work in high wind velocity without sustaining critical damage that could lead to the fracture of the device^18^,^19^,^20^,^21^,^22^,^23^. The creation of the wind flow in the device needs both an inlet and outlet to make a path for the wind flow. A throat with a constricted area less than the inlet functions as the nozzle inside the WR-TENG to increase the wind velocity. In the event when wind is blown to an inlet, the Venturi effect causes the wind velocity to increase along the path leading from the inlet to the throat. Although real fluids including air are compressible flows with significant changes in fluid density, the compressible flows are considered as incompressible flows when the Mach number is smaller than 0.3 (air flow velocity < 102 m/s) by empirical practices. Therefore, the incompressible fluid characteristics follow Equations (1) and (2) as given below:


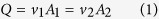



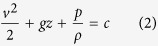


where *Q* is volume flow rate, *A* is cross-sectional area, *v* is wind velocity, *g* is the gravity acceleration, *z* is the elevation of the point, *p* is the pressure, *ρ* is the density of the fluid, and c is a constant value. In order to consider the fluid velocity at the inlet and after the throat, the fluid characteristic at the nozzle satisfies the following Equation (3):


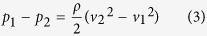


Hence, the flow velocity increases due to the principle of mass continuity when wind passes through the throat. This decreases its static pressure in accord with the conservation of mechanical energy principle. Figure 1b shows photographs of the EPS sphere movement when the air flow velocity is 2.2 m/s. As shown in the photographs, the EPS sphere rotates inside the vortex whistle cylinder. The wind flow velocity increases as the wind passes through the throat, and the direction continuously changes due to the cylinder packaging. Thus, the ambient wind turns into a rapid vortex flow in the cylindrical vortex whistle creating the rotating orbit for the EPS sphere.

In the WR-TENG, a negatively charged EPS sphere rotates over positively charged Ag electrodes printed on the inner surface of the WR-TENG. As shown as Fig. 1c, two motions of the EPS sphere, namely rolling motion, and bouncing motion explain the working mechanism of the WR-TENG. The drag force created as the wind rotates through the vortex whistle causes the EPS sphere to move along the wind flow. This drag force induces the rolling and rotating motions of the EPS sphere inside the cylinder. In the rolling mode, the negatively charged EPS sphere contacts with the silver electrode as it rolls through the inner surface of the cylinder, and reaches an electrical equilibrium by gaining electrons from the ground in a single-electrode mode^21^ or from the connected electrode in a freestanding mode^22^. After the EPS sphere passes through the silver electrodes, an electrical potential difference is generated on the silver electrode and electrons flow back to the ground or to other connected electrodes. Thus, alternative current is generated due to a continuous contact-separation of the EPS sphere during the rolling motion. Conversely, the EPS sphere separates from the inner surface of the cylinder when it reaches the throat due to shape of the throat and the high-speed wind flow passing through the nozzle. Following the separation of the EPS sphere, it bounces on the inner wall of the cylinder until the sphere is stabilized on the rotating orbit. An alternative current is also generated by the contact-separation between the EPS sphere and Ag electrodes due to the bounce motion.

**Figure 2:**
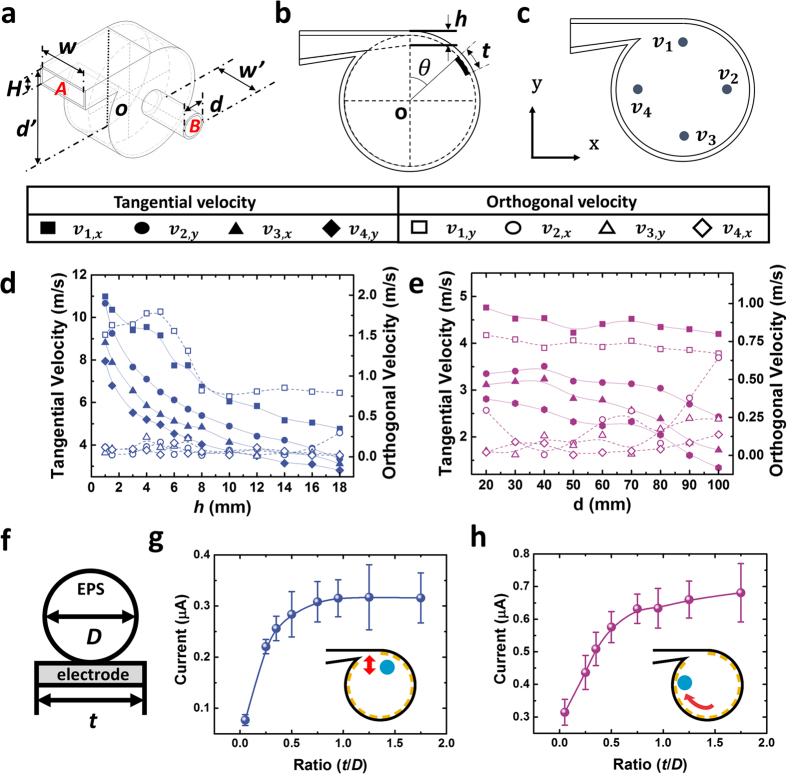
Computational fluid dynamics results of the WR-TENG and output based on the sphere-electrode size ratio. (**a**) A 3-D model used in the simulation, and (**b**) Various parameters used in the optimization. (**c**) The wind velocity measured points using CFD. (**d**) Tangential and orthogonal wind velocities calculated depending on the height of the throat, and (**e**) the outlet diameter. (**f**) The simplified structure and parameters used in the sphere-electrode size ratio. (**g**) Bouncing, and (**h**) rolling *I*_*CC*_ outputs based on the sphere-electrode size ratio.

Although the wind flow is an essential parameter for the EPS sphere to rotate along the vortex whistle, the unconstrained nature of the wind flow makes it hard to analyze in complex designs. Therefore, the wind vortex flow is visualized by computational fluid dynamics (CFD) software and the substrate of the vortex whistle is optimized by the simulation results. The 3-D model used in the CFD is illustrated in Fig. 2a,b and its parameters are listed in Table 1. In the simulation, the wind velocity is measured at the points *v*_1,_ *v*_2,_ *v*_3_ and *v*_4_ as shown in Fig. 2c. The direction of tangential and orthogonal velocities at each point is illustrated in Figure S1 (Supplementary information). Given that the wind goes through the vortex whistle and that the EPS sphere is pushed by the rotating wind, the drag force acting upon the EPS sphere is the main cause of the EPS sphere rotation along the inner cylinder. The drag force could be explained by Equation (4) given below:


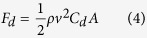


where *F*_*d*_ is the drag force, *ρ* is the density of air, *v* is relative velocity between the object and the air, *C*_*d*_ is the drag coefficient, and *A* is the cross-sectional area. As shown in Equation (4), the wind velocity is a crucial factor in understanding the motion of the EPS sphere inside the WR-TENG. As the performance of the general TENG is known to improve in high frequency movements in both vertical contact-separation and sliding TENGs^24^,^25^, the velocity of the EPS and the stability of the EPS movement orbit is examined through the simulation. Figure S2a (*h* = 8 mm, *d* = 20 mm) visualizes the flow in the WR-TENG, and it indicates that the air is blown to the inlet, rotates along the wall forming vortex flow, and then exits through the outlet. Specifically, the tangential wind velocity is the main parameter for the accelerating EPS sphere, and the orthogonal wind velocity is a factor that disturbs the orbital motion of the EPS sphere. In order to determine the effect of the two factors, the tangential and orthogonal wind velocities are calculated depending on various heights of the throat *h* and the diameters of outlet *d* though the CFD analysis. As shown in Fig. 2d,a reduction in *h* increases the tangential velocity due to the Venturi effect while air flows through the nozzle. With respect to the orthogonal velocity, the points *v*_2_,*v*_3_,*v*_4_ indicate almost no change in orthogonal velocity, but the orthogonal velocity of point *v*_1_ tends to be higher than other points, particularly until *h *= 8 mm. This is because the point *v*_1_ is located near the entrance where the slope of the bottom surface of the inlet forms a nozzle shape increasing the wind flow velocity.

The wind flow converges to the top of the whistle surface due to the nozzle shape, and spreads out to a large cylindrical cavity disturbing the stability of the EPS sphere rotation orbit (Figure S2b). As shown as Figure S2b–ii, iii, increasing the orthogonal wind velocity increases the disturbance of the EPS sphere rotation orbit when the height of the throat *h* is smaller. At points *v*_2_,*v*_3_,*v*_4_, the wind flow stably forms a vortex orbit while moving along the inner surface of the vortex whistle. The location of the outlet is also considered to form a proper vortex flow. There is interference in the rotation orbit of the EPS sphere if the outlet is located on the side of the cylinder substrate due to the air exiting from the cylinder and the disturbance of the rotation orbit caused by the outlet itself (Figure S3a). Therefore, in order to form a fully developed vortex flow, the outlet of the WR-TENG is located on the central axis of the cylindrical substrate. In Fig. 2e, the inner diameter of the outlet *d* is analyzed from 20 mm to 100 mm depending on the CFD for optimization. In this simulation, *h* is fixed at 16 mm that equals the height of the inlet *H*. As the inner diameter of the outlet *d* increases, the tangential velocity at the points *v*_1_,*v*_2_,*v*_3_,*v*_4_ decreases because the flow exits through the outlet before it forms a fully developed vortex (Figure S3b). The size ratio between the electrode and dielectric material is an important factor for the space-efficiency design. The dielectric material used in the WR-TENG has a spherical shape where the electrical performance efficiency depends on the ratio of the Ag electrode width *t* (1~35 mm) and the diameter *D* of the EPS (20 mm) (Fig. 2f). The electrical performance of the WR-TENG depends on the sphere-electrode size ratio as illustrated in Fig. 2g,h. Both the outputs increase continuously until the size ratio reaches 0.5, and then they gradually saturate owing to the spherical shape of the dielectric material. Furthermore, the size of the EPS sphere is the one of the most important factor for the performance of device. In general case, the maximum diameter ratio is 0.2 and its detailed in Supplementary Information, Section 2-1.

**Figure 3:**
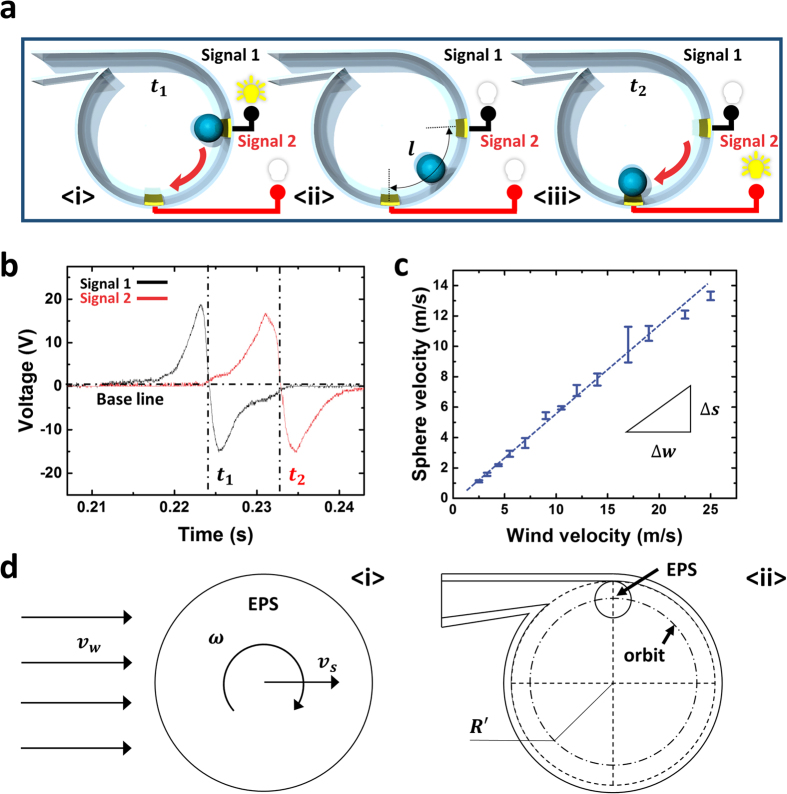
The WR-TENG used as a self-powered wind velocity sensor. (**a**) A schematic illustration of the WR-TENG wind velocity sensor. (**b**) The *V*_*OC*_ output generated at points t_1_ and t_2_. (**c**) The relationship between the calculated sphere velocities and the measured wind velocities. (**d**) A simplified illustration and various parameters used to calculate the kinetic energy of the EPS sphere.

The CFD results showed the wind sensitive characteristics of WR-TENG indicating that it could be designed as a self-powered anemometer. The relationship between the wind flow velocity and angular velocity of EPS sphere rotation was used as a wind velocity sensing parameter (Fig. 3a) as the rotation of the EPS sphere was driven by the wind. The center of the Ag electrodes was located at the point 90° (*v*_2_) and 180° (*v*_3_) from the top of the cylinder with low orthogonal wind velocities (Fig. 2d,e). The ratio of the EPS sphere and the Ag electrode used to fabricate the optimized WR-TENG was 0.75 (diameter *D* = 20 mm, width *t* = 15 mm) where the *I*_*CC*_ output was saturated (Fig. 2g,h). Signal 1 was generated at *t*_1_ when the negatively charged EPS sphere moved on the first electrode (Fig. 3a–i). Then, the EPS sphere rolled along the rotating orbit until distance *l*, and the center of the sphere moved from electrode 1 to 2 (Fig. 3a–ii). Signal 2 was generated at *t*_2_ when the sphere reached electrode 2 (Fig. 3a–iii). Both electrode 1 and electrode 2 are single-electrode mode TENGs that are connected to a ground. As shown in Fig. 3b, the output generated in each electrode has points *t*_1_ and *t*_2_ where the wave form reaches 0 when the electric charge reverses direction. Therefore, using the distance between the electrode *l* and time *t*_1_ and *t*_2_ in the rotating motion, the sphere velocity *v*_*s*_ could be determined by the following Equation (5):


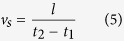


The plot between the measured wind velocity and the calculated sphere velocity according to this equation is illustrated in Fig. 3c, and the measured output based on wind velocity is depicted in Figure S4. As shown in the plot, the wind velocity and the sphere velocity have a linear relation. The experimental converting constant *C* indicates the relationship of the sphere velocity *v*_*s*_ and the wind velocity *v*_*w*_ as expressed in Equation (6) given below:


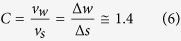


The linearity is a suitable characteristic for the wind velocity sensor where the signals of sphere velocity could be directly interpreted as wind velocities. Additionally, the WR-TENG could be used for a wide range of wind velocities because of its high sensitivity (*C* = 1.4) due to the lightweight dielectric material.

The most influential factor for wind energy harvesting is the kinetic energy of the EPS sphere converted from wind energy. Therefore, the efficiency of converting wind energy to kinetic energy in the WR-TENG is derived by the relation between the sphere and wind velocity. The kinetic energy of the EPS sphere is an essential parameter for the high efficiency of the WR-TENG. The parameters utilized to calculate the efficiency are illustrated in Fig. 3d and Table 2. As discussed previously, the sphere velocity and wind velocity share a linear relationship between sensing points *v*_2_ and *v*_3_ when the wind continuously blows. Figure S5 shows that the average of the tangential wind velocity *v*_*s*_ at each point is between points *v*_2_ and *v*_3_. Therefore, a converting constant *C* could be applied while the EPS sphere is rotated through the inner cylinder. The movement of the EPS sphere when the wind blows at a velocity of *v*_*w*_, could be expressed as moving at a velocity of *v*_*s*_ and rotating at an angular velocity of *ω* (Fig. 3d–i). The kinetic energy of the EPS sphere could be expressed as the sum of the translational and rotational kinetic energies about the center of the mass Equation (7) given below:


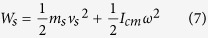


The EPS sphere moves along the orbit of radius *R*′ (Fig. 3d–ii). Given that the EPS sphere gains kinetic energy through wind at *v*_*w*_ for the time *t* required for a rotation cycle, the work performed by the wind could be expressed according to Equation (8) as follows:


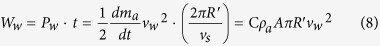


Based on Equations (7) and (8), the efficiency of converting the wind energy to kinetic energy could be expressed as Equation (9) (a detailed derivation is shown in Supplementary Information, Sections 2-2):


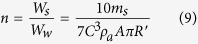


According to the results, the WR-TENG indicates a high efficiency (38.28%) of converting wind energy to kinetic energy. Furthermore, the magnitude of the wind velocity is an independent parameter from the efficiency of converting wind energy to kinetic energy, and this suggests the availability of the wind sensor and generator for a wide range of wind velocities with a high efficiency.

**Figure 4:**
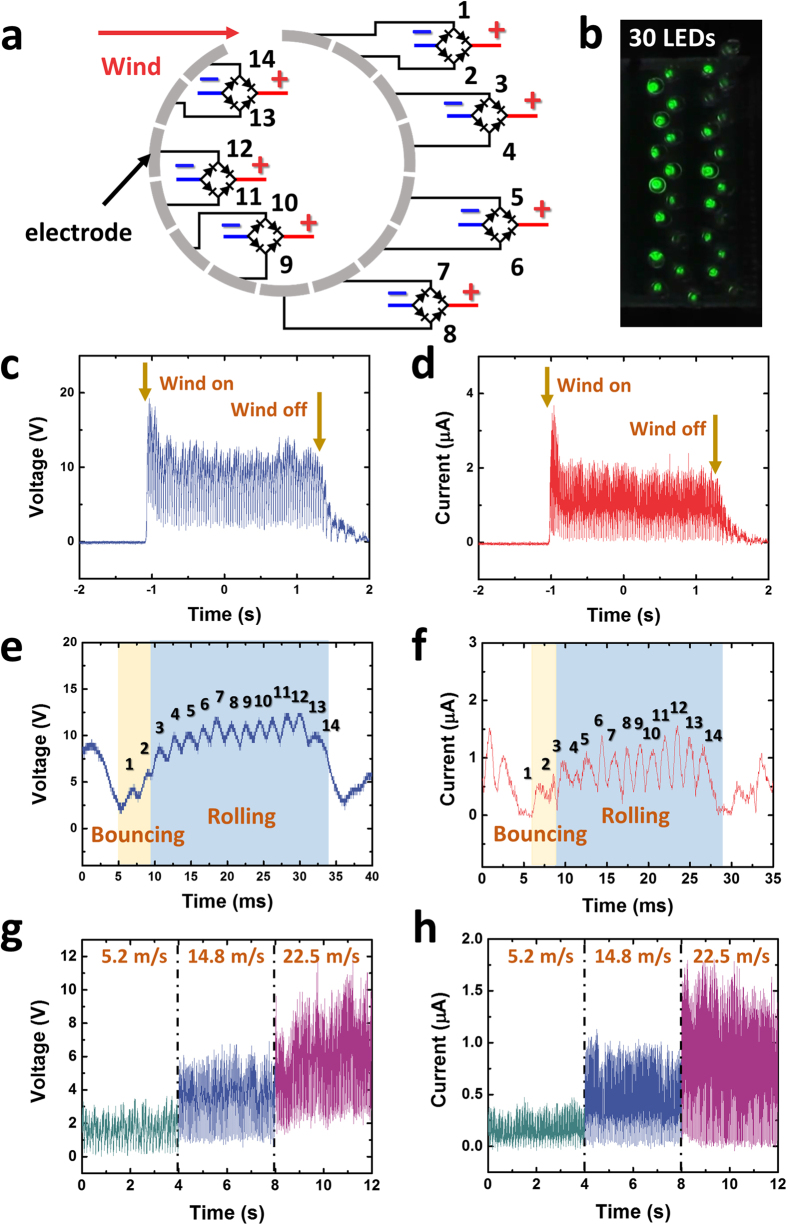
The WR-TENG as a wind energy harvester with multiple units. (**a**) A patterned electrode connected as a free-standing mode TENG. (**b**) The WR-TENG lighting 30 LEDs. The (**c**) *V*_*OC*_, (**d**) *I*_*CC*_ output, and (**e**) magnified *V*_*OC*_, (**f**) *I*_*CC*_ output of a multiple patterned WR-TENG at wind velocities of 22.5 m/s. The (**g**) *V*_*OC*_ and (**h**) *I*_*CC*_ outputs of a multiple patterned WR-TENG based on wind velocities.

Figure 4a shows the patterned electrode of the WR-TENG with 14 electrodes aligned along the inner cylinder used in the experiment. Every two electrodes along the cylinder are connected as a unit of the free-standing mode TENG. The free-standing pattern electrodes were used to increase the output of the WR-TENG when compared to a single-electrode mode as shown in Figure S6. Each free-standing generating unit was connected to a rectifier to show a positive output and to avoid any cancellation of electrical outputs caused by a phase difference between each unit. The integrated WR-TENG could light up 30 LEDs instantaneously when wind was blow at 22.5 m/s (Fig. 4b, Video S1 in Supplementary information). Figure 4c,d indicates open-circuit voltage (*V*_*OC*_) and closed-circuit current (*I*_*CC*_) of the multiple patterned WR-TENG at a wind velocity of 22.5 m/s. As shown in the plot, the WR-TENG generates electrical power output when the wind was blown. The high peak point when the wind was blown initially was due to a sudden pressure change in the wind flow. Following this, the output of the WR-TENG was stabilized. The average *V*_*OC*_ and *I*_*CC*_ of the generated output peaks were 8.05 V and 1.59 μA, respectively. Despite the ceasing of the wind, the EPS sphere continued to rotate inside the vortex whistle and generate additional output because of its inertia. Figure 4e,f illustrates a magnified plot of *V*_*OC*_ and *I*_*CC*_ generated by the WR-TENG. As the EPS sphere rotated along the inner surface of the vortex whistle, the sphere contacted and separated with every electrode in the whistle. As shown in both the figures, there are 14 peaks generated by each electrode fabricated inside the cylinder. As discussed previously, the mechanism of the WR-TENG could be divided into the rolling motion caused by the wind flow and the bouncing motion near the whistle inlet. The first two output peaks shown in Fig. 4e,f were generated through the small bouncing motion of the EPS sphere. The electrical output for the bouncing motion was mainly dependent on the amount of the kinetic energy of the dielectric materials. In the bouncing region, the EPS sphere bounced off from the inner cylinder due to the inlet and the incoming wind flow. With respect to the kinetic energy perspective, the EPS sphere instantaneously loses kinetic energy causing a decrease in the velocity when it bounced on the inner surface (electrodes 1–2). In the rolling region, the sphere gained higher kinetic energy as the wind pushed the EPS sphere to roll around the inner cylinder and generated a higher output (electrodes 3–14). Hence, the electrical output of the vertical contact-separation motion was smaller when compared to the average output of the EPS sphere rotation cycle. As the wind velocity was the most important factor, Fig. 4g,h shows the *V*_*OC*_ and *I*_*CC*_outputs of the WR-TENG according to the wind velocity. The velocity of the rotating ball increased the centrifugal force that the EPS sphere received in the cycle causing an increase in the effective contact area^26^,^27^. Therefore, both *V*_*OC*_ and *I*_*CC*_ increased as the wind velocity increased. Figure S7 is a plot of the voltage and current output based on the resistance, and the power output according to the voltage and current output. The electrical power output measured in the plot was based on a single unit inside the WR-TENG. The multiple patterns inside the WR-TENG generated multiple outputs in a single rotation of the EPS sphere that could multiply the total power generated in the WR-TENG by the number of generating units inside.

**Figure 5:**
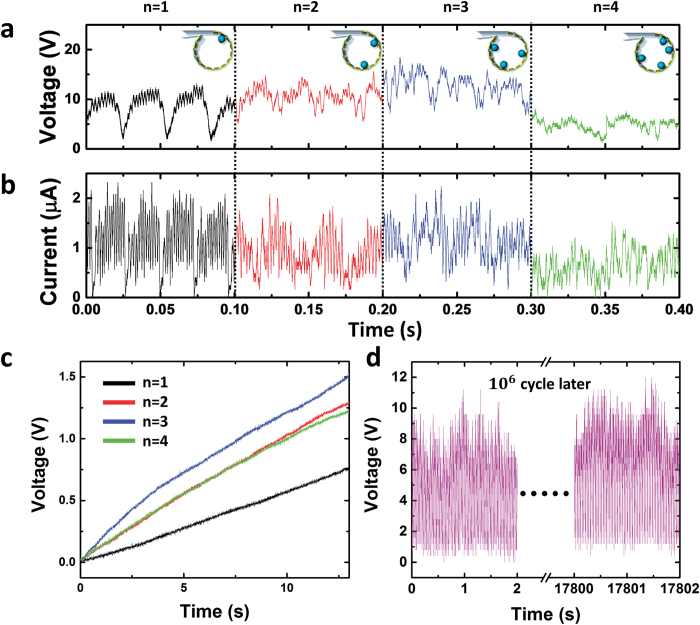
Electrical power output of the WR-TENG based on the number of EPS spheres and the durability of the WR-TENG. The (**a**) *V*_*OC*_, and *I*_*CC*_ outputs of the WR-TENG based on the number of EPS spheres inside the vortex whistle. (**c**) A 100 μF capacitor charging rate depending on number of spheres inside. (**d**) The durability test (>10^6^ cycle) results for the WR-TENG.

The number of EPS spheres could also be a significant factor for the WR-TENG in case of generating multiple outputs. In Fig. 5a,b, the *V*_*OC*_ and *I*_*CC*_ outputs of multiple EPS spheres in the WR-TENG are shown. Every two electrodes were connected as a free-standing mode similar to that detailed in the previous paragraph. In the case when there was only one EPS sphere rolling inside the WR-TENG, the peaks formed a uniform wave pattern due to the output reduction on the wind flow entrance. As the number of spheres increased, the output of the WR-TENG increased accordingly because the electrical power peak generated from each EPS sphere converged and formed higher peaks. As shown in the plot, both the *V*_*OC*_ and *I*_*CC*_ outputs tended to have more electrical power peaks until there were 3 EPS spheres inside the cylinder. After 4 EPS spheres were inside the WR-TENG, the EPS spheres collided with each other and disturbed the rotating orbit themselves. The kinetic energy of each EPS sphere decreased when the spheres collided and the output of the WR-TENG decreased. Figure 5c shows the WR-TENG charging a 100 μF capacitor based on number of balls inside. As shown in the plot, 3 spheres inside the WR-TENG indicated the highest charging rate. Although a single sphere output of the WR-TENG had a higher output than the 4 spheres inside, the number of peaks generated in a single cycle was lower than 4 spheres causing a single sphere to have the lowest rate in charging a capacitor.

In order to measure the durability of the WR-TENG, the amount of the centrifugal force applied by the EPS sphere for a wind velocity of 22.5 m/s is expressed as Equation (10) given below:


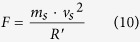


According to the parameters listed in Table 3, the calculated *F* was 1.01 N. Although the initial contact of EPS sphere and electrode was a dot contact, it contacted the Ag electrode as a circular shape due to the centrifugal force applied on the EPS sphere. The radius of the circle *a* contacted by the EPS sphere with the Ag electrode can be expressed as Equation (11) as follows:


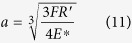


where, E^*^ follows Equation (12) given below:


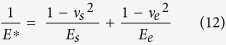


The calculated *a* with the parameters of the WR-TENG was 0.09357 mm. The maximum contact pressure occurred in the center of circle, and it could be expressed as Equation (13):


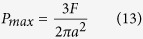


Given the calculated *F* and *a*, the maximum contact pressure caused by the EPS sphere was 552.16 kPa. The practical use of the WR-TENG in order to avoid the loss of function necessitates considering the factor of safety, which is a term for the capacity to allow for uncertainties. The factor of safety can be described as Equation (14) given below:


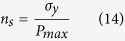


The safety factor for the WR-TENG is 99.61 considering that yield strength of silver is 55 MPa. With respect to the EPS perspective, deformation occurred at the outer surface of the sphere that was in contact with the inner surface of the vortex whistle since the yield strength of the EPS was 208.23 kPa. The contact surface area increased as the deformation occurred, and the maximum contact pressure caused by the centrifugal force decreased. Additionally, there were elastic, plateau, and densification regions without fracture over the yield strength when the EPS was compressed^28^,^29^,^30^. Therefore, there was no fracture or critical damage even if the EPS spheres deformed due to the contact pressure. Furthermore, even after 10^6^ cycles of rotation, the WR-TENG showed a steady output as illustrated in Fig. 5d. This result indicated that the design of the WR-TENG was highly reliable for practical applications.

## Conclusion

In summary, a novel wind-driven TENG-based was developed on a vortex whistle. The WR-TENG indicated wind flow sensitive characteristics due to the nozzle design and the light-weight dielectric material that could be utilized as both a self-powered wind velocity sensor and a wind powered generator. The wind velocity could be calculated by using a converting constant *C* that uses the linear relationship of the sphere and wind velocity in the cylinder. The efficiency of converting the wind energy to the kinetic energy of the EPS sphere was shown as approximately 40%. A multiple patterned electrode was fabricated to increase the output efficiency in a single cycle. Moreover, optimized results according to the number of spheres were examined. Theoretical analysis and experimental results indicate a generating output without degradation even after 1 million cycles at wind velocity of 22.5 m/s and a safety factor of 99.61, thereby indicating that the design of the WR-TENG is reliable for practical applications. This study demonstrates a novel approach for developing the performance of sensitive and robust wind-driven TENGs, and a practical method for utilizing wasted wind energy.

## Methods

### Fabrication of the Wind-rolling TENGs

The polyvinyl chloride (PVC) substrate of the WR-TENG with a thickness of 300 μm was used to control the wind flow to convert wind energy to kinetic energy with its enclosed design. The PVC sheet was attached without a gap to form an inlet and outlet of the vortex whistle. A 20 mm diameter EPS sphere was placed inside the whistle, and it rotates around the inner surface of the vortex whistle when wind is blown through the whistle. The silver paste (AgIC conductive ink) electrodes that is printed on PET sheet by circuit printer were patterned on the inner surface of whistle cylinder. For enhanced reliability of WR-TENG, the electrodes were located the specific point like Figs 3a and 4a.

### Analysis of the vortex flow by Computation Fluid Dynamics (CFD)

In this study, for optimization, the flow in vortex whistle was analyzed by Computation Fluid Dynamics (Autodesk CFD). Utilized materials PVC is adapted on vortex whistle and inside is full of air. For the setting boundary condition, two section inlet ***A*** and outlet ***B*** were selected with the properties is illustrated in Fig. 2a and Table 1. All of the result of point *v*_*k*_(k = 1 to 4) has the same constant that middle surface of cylinder.

### Electrical Measurement of the TENGs

The output voltage and current signals were measured by a digital oscilloscope (Tektronix MDO3012) and a current preamplifier (Stanford Research Systems SR570).

## Additional Information

**How to cite this article**: Yong, H. *et al*. Highly reliable wind-rolling triboelectric nanogenerator operating in a wide wind speed range. *Sci. Rep.* **6**, 33977; doi: 10.1038/srep33977 (2016).

## Supplementary Material

Supplementary Information

Supplementary Video S1

## Figures and Tables

**Table 1:** Parameters utilized in the CFD analysis of the WR-TENG.

Height of the inlet *H*	16 mm
Width of the inlet *w*	50 mm
Inner diameter of the cylinder *d*′	100 mm
Length of the outlet *w*′	50 mm
Inner diameter of the outlet *d*	20 mm
Boundary condition at *A*	5 m/s (velocity)
Boundary condition at *B*	0 kPa (pressure)
Position at *v*_*k*_	(*r*, *θ*) = (40, (*k−*1) 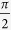 )

**Table 2:** Parameters utilized in the derivation of the kinetic energy conversion efficiency.

Weight of EPS sphere *m*_*s*_	0.08 g
Density of air *ρ*_*a*_	1.225 kg/m^3^
Cross sectional area of EPS sphere *A*	314.16 mm^2^
Radius of EPS sphere’s orbit *R*′	40 mm

**Table 3:** Parameters utilized in the safety factor by Hertz’s spherical contact.

Young’s modulus of silver *E*_*s*_	83 GPa
Young’s modulus of EPS *E*_*e*_	4070 kPa
Poisson ratio of silver *ν*_*s*_	0.37
Poisson ratio of EPS *ν*_*e*_	0.11
Yield strength of silver *σ*_*y*_	55 MPa
