# Supplementary material for: Highly reliable wind-rolling triboelectric nanogenerator operating in a wide wind speed range
Source: Sci Rep. 2016 Sep 22;6:33977. doi: 10.1038/srep33977 (PMC5032013; doi:10.1038/srep33977)
Supplement: Supplementary Information [file srep33977-s1.doc]

**Highly reliable wind-rolling triboelectric nanogenerator operating in a wide wind speed range**

**Hyungseok Yong**^1^**,**+**, Jihoon Chung**^1^**,**+**, Dukhyun Choi**^2^**, Daewoong Jung^3^, Minhaeng Cho**^1^**^,*^, and Sangmin Lee**^1^**^,*^**

^1^School of Mechanical Engineering, Chung-Ang University, 84, Heukseuk-ro, Dongjack-gu, Seoul 156-756, Republic of Korea

^2^Department of Mechanical Engineering, College Engineering, Kyung Hee University, 1732, Deogyeong-daero, Giheung, Yongin, Gyeonggi 446-701, Republic of Korea

^3^Aircraft System Technology Group, Korea Institute of Industrial Technology (KITECH), Yeongcheon-si, Gyeongbuk-do, 38822 Republic of Korea^*^corresponding.

Prof. M.Cho. E-mail: mhcho87@cau.ac.kr.

Prof. S. Lee. E-mail: slee98@cau.ac.kr. Homepage: http://slee.cau.ac.kr

^+^these authors contributed equally to this work

**Section 1**


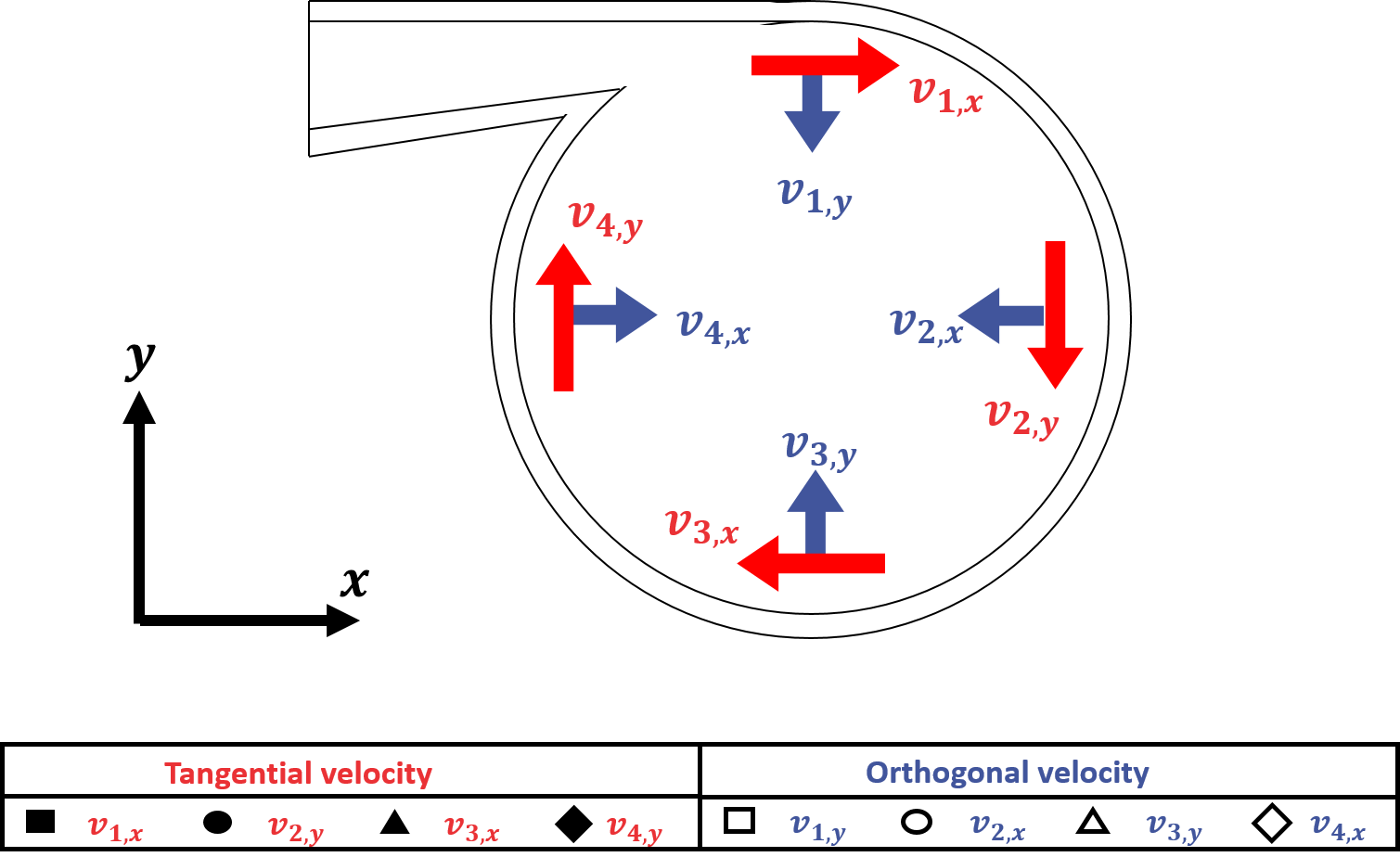


**Figure S1.** The direction of the tangential and orthogonal velocities at each measured point.


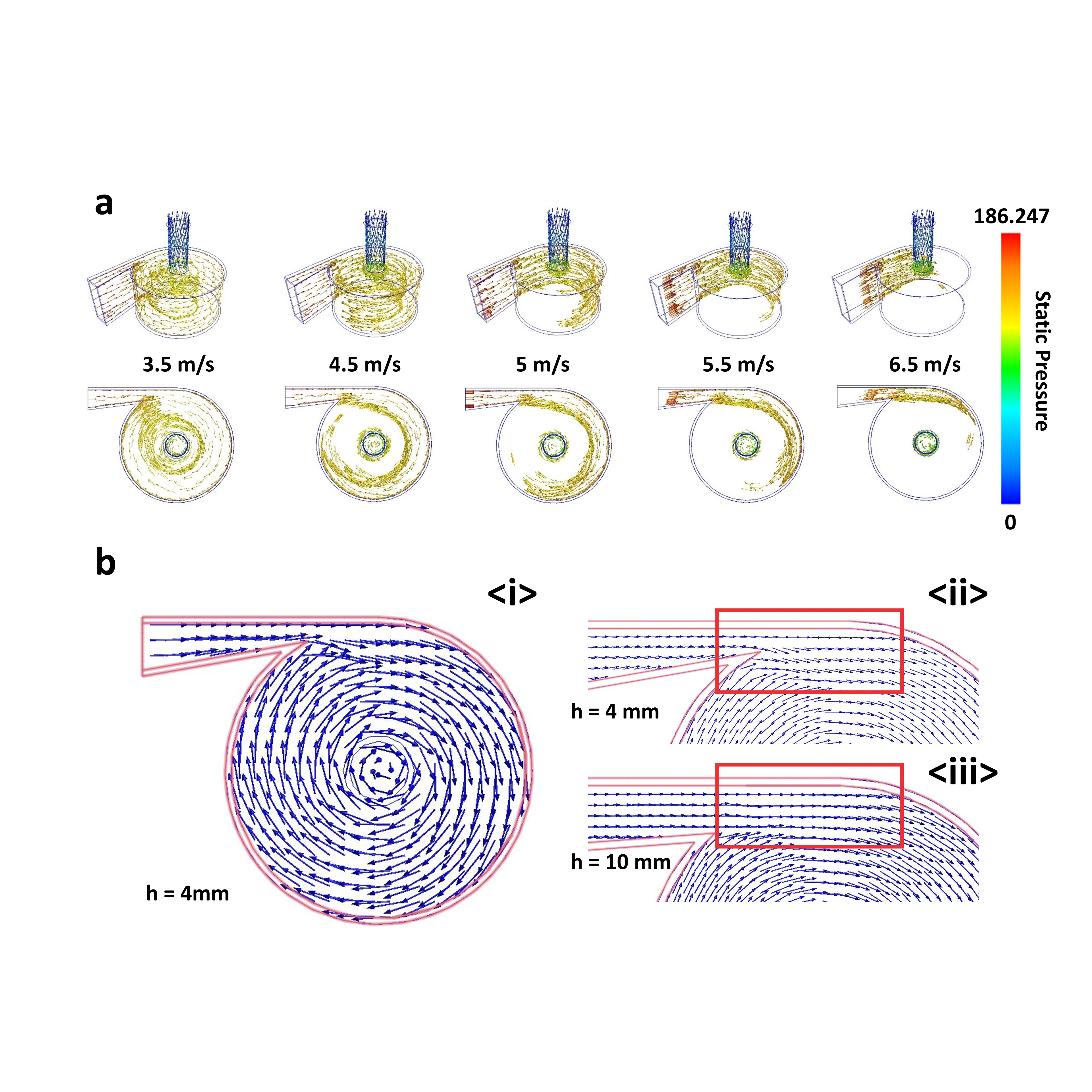


**Figure S2.** CFD analysis in various conditions. (a) The vortex flow formed inside the whistle for various wind velocities. (b) The influence of the nozzle shape throat on wind velocities in the vortex whistle.

**
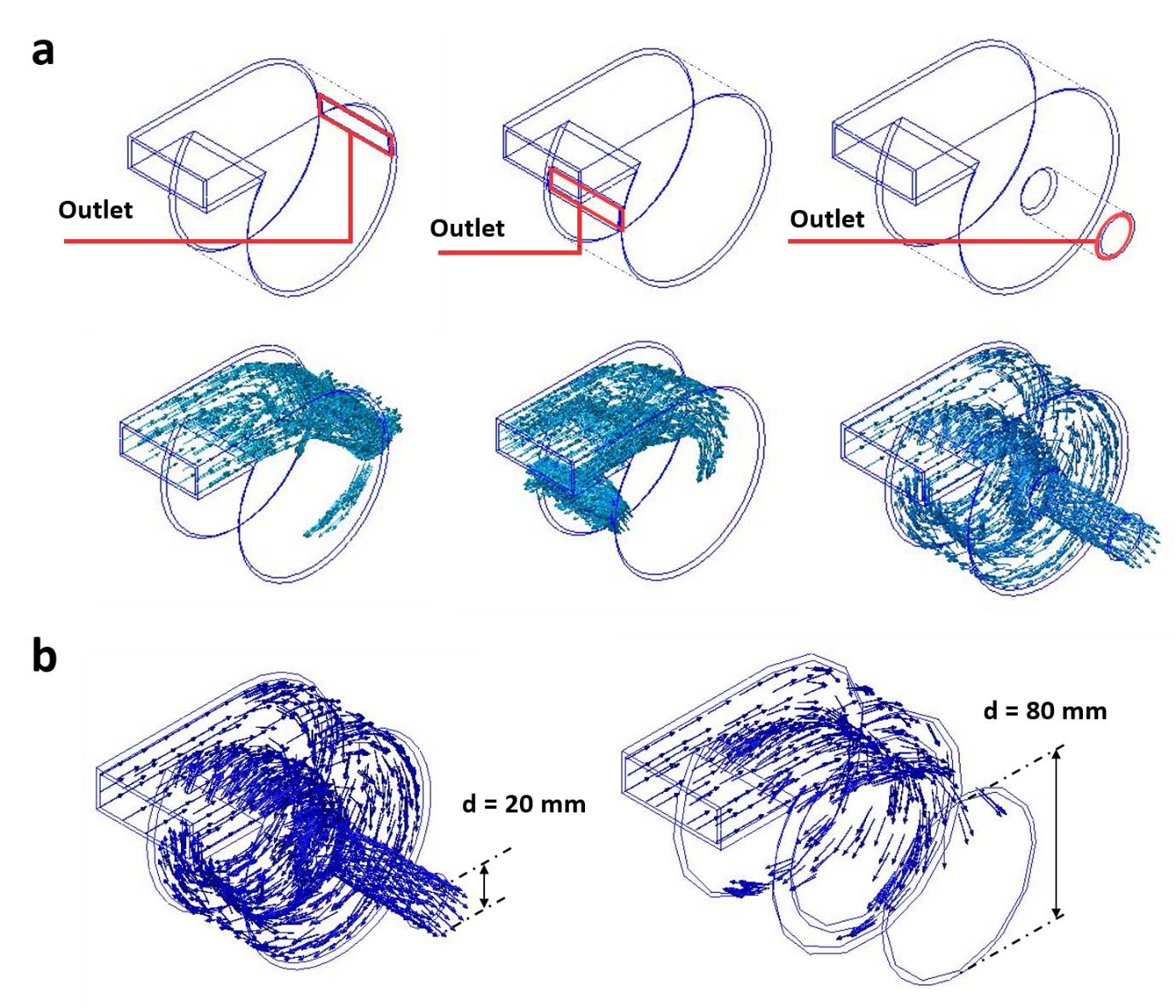
 Figure S3.** CFD analysis in various conditions. (a) The influence of the outlet location, and (b) the outlet size on wind flow inside the whistle.


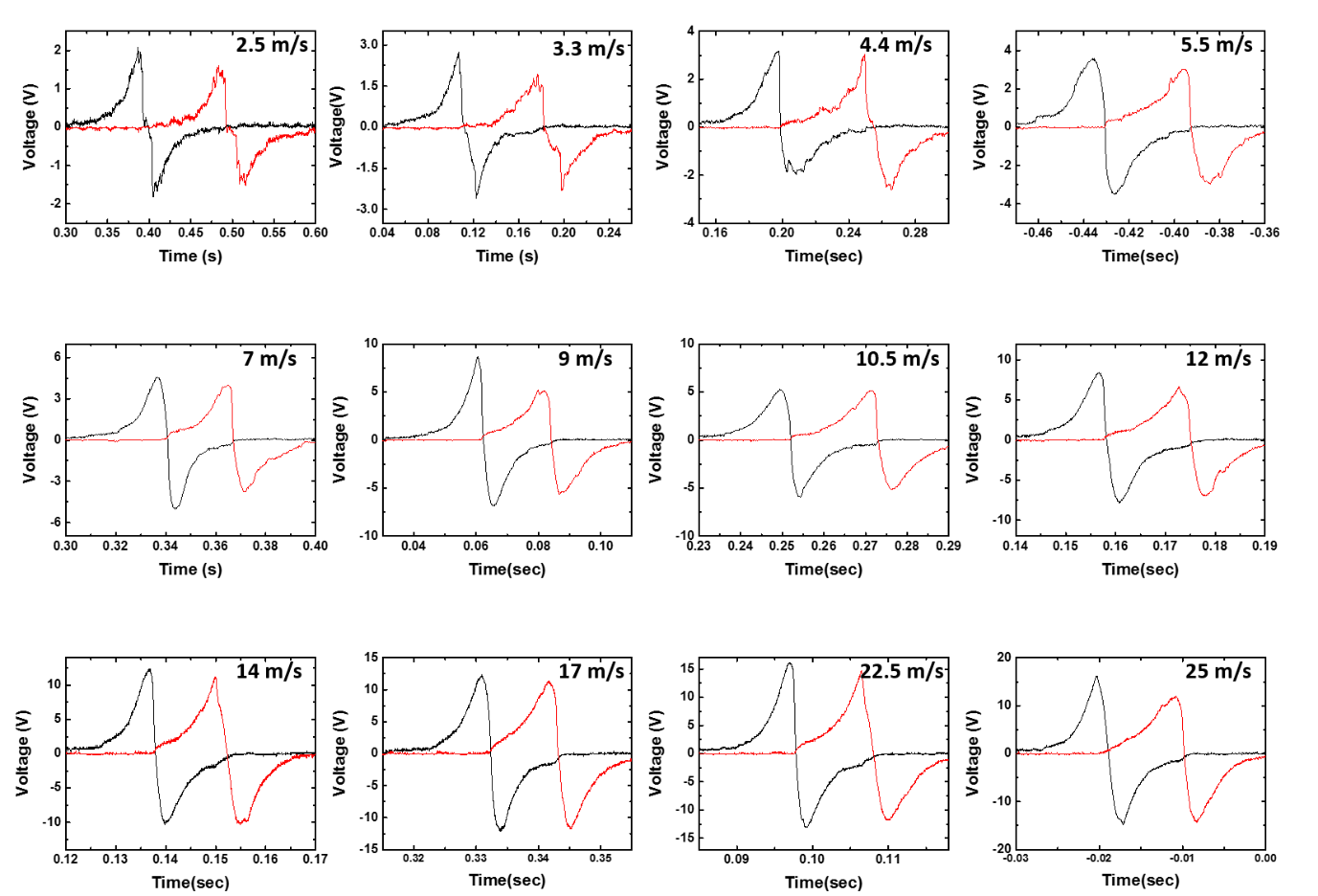


**Figure S4.** Measured *V_OC_* outputs based on various wind velocities at points
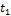
, and
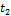


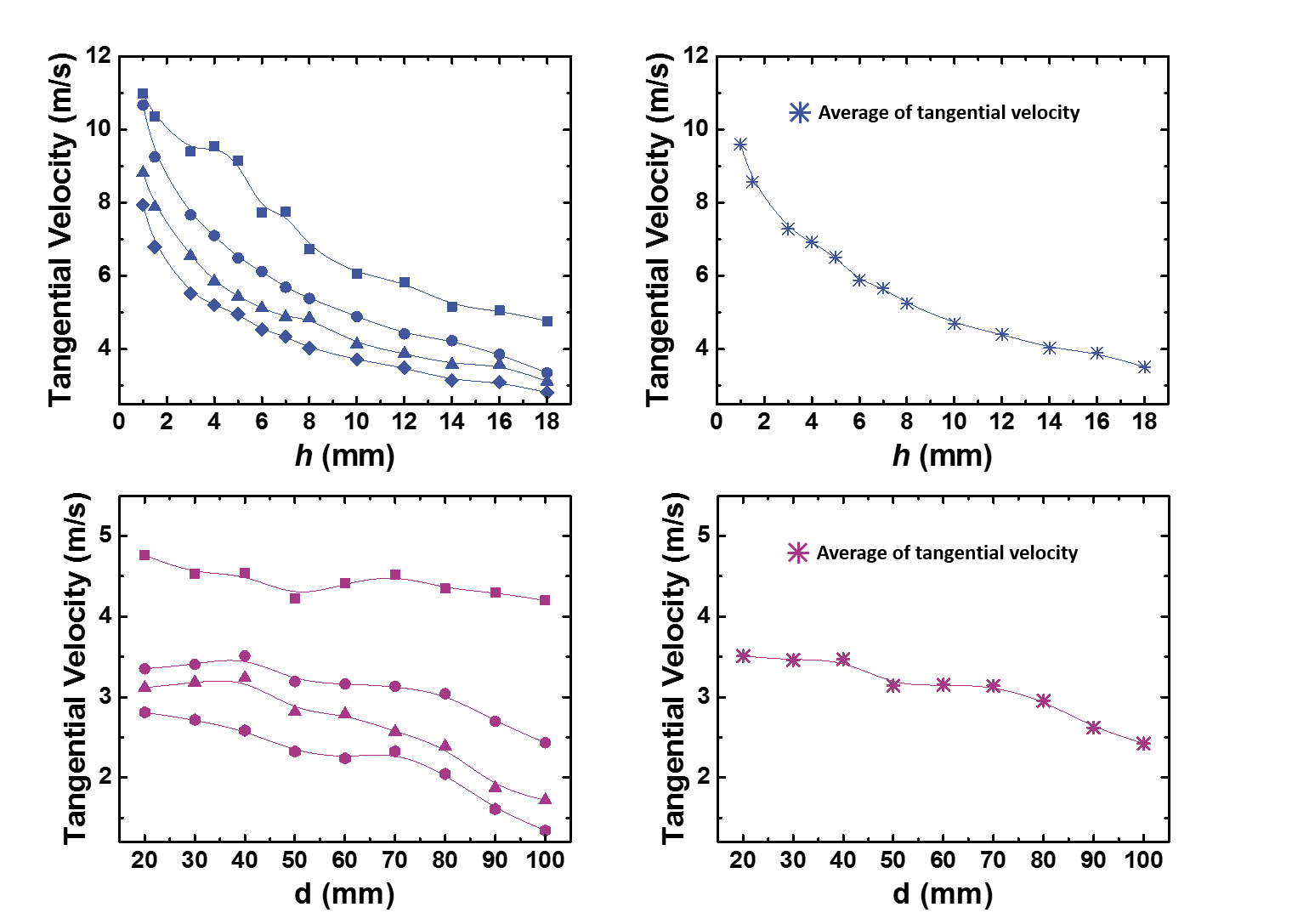


**Figure S5.** Average tangential wind velocities plotted at points
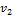
, and
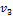
.


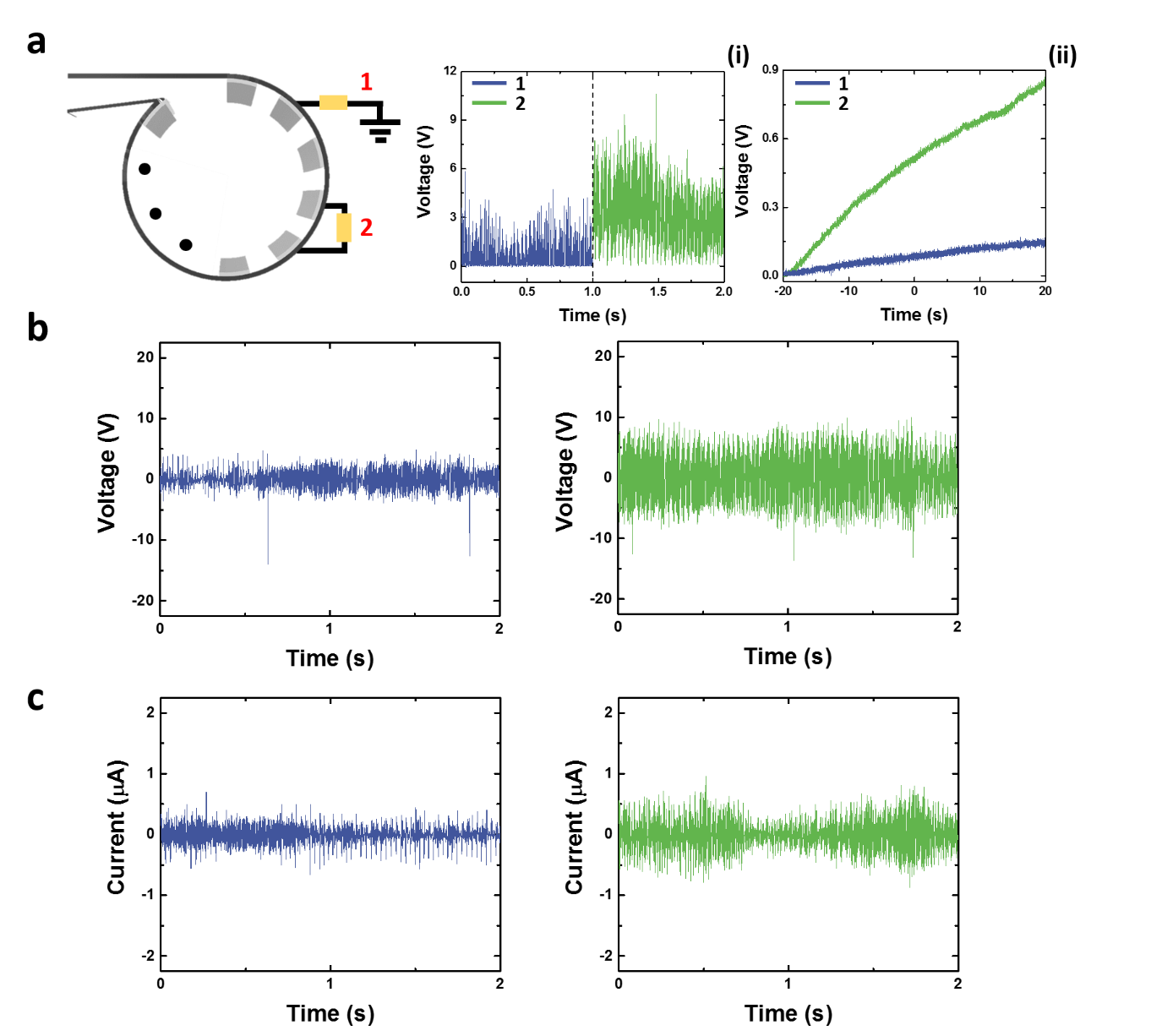


**Figure S6.** Output comparison between a single-electrode mode TENG and a free-standing mode TENG. (a) A simplified illustration of the WR-TENG, (i) voltage, (ii) 100 μF capacitor charging rate comparison between a single-electrode mode and a free-standing mode TENG. The (b) *V_OC_* and (c) *I_CC_* comparison between a single-electrode mode and a free-standing mode TENG.


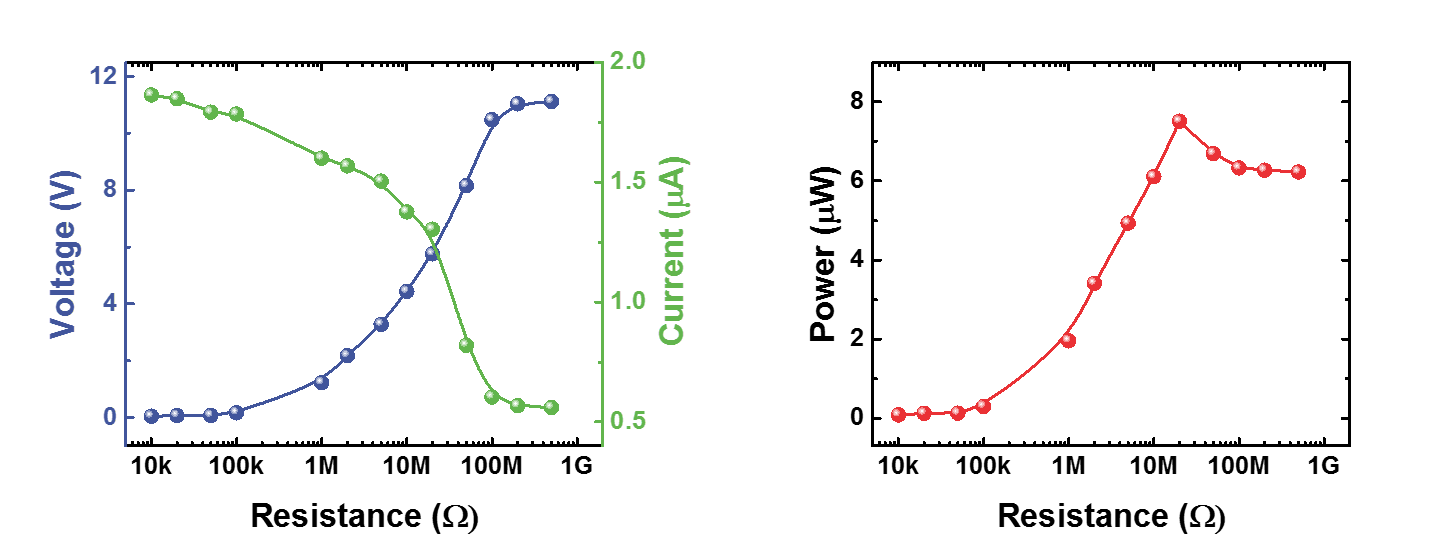


**Figure S7.** (a) The dependence of voltage, current, and power on the value of the external load resistance.

**Section 2 – 1**


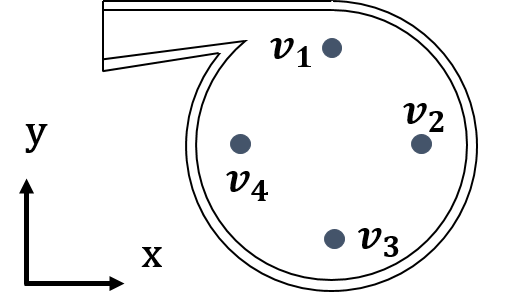
The size of the EPS sphere is the one of the most important factor for the performance of device. The tangential velocity that accelerates EPS spheres is a factor that stables orbital motion of the EPS sphere, and the orthogonal velocity is a factor that disturbs the stable motion. These two factors can be used as standard design factors for WR-TENG.

In the simulation, the wind velocity distributions are measured at the points v_2_ in above figure, and the average of tangential velocity and orthogonal velocity were measured as the figure below where distance means the distance from the center of cylinder (radius of the cylinder = 50 mm).


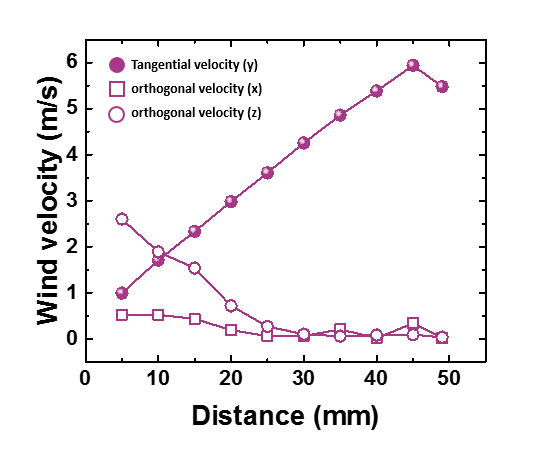
 As shown in the plot, the tangential velocity is the lowest when the measure point is near the center. The sudden decrease after 45 mm distance is due to no slip condition near the cylinder substrate surface. On the other hand, the orthogonal velocity gradually decreases until 30 mm distance and the value is near 0 after. Therefore, as the orthogonal velocity is a factor to be avoided, the maximum diameter ratio between the EPS sphere and the cylinder is where the orthogonal velocity is the lowest. In general case, the maximum diameter ratio is 0.2.

**Section 2 – 2**

Derivation of Equation 7, Equation 8, and Equation 9 for the efficiency of converting wind energy to kinetic energy


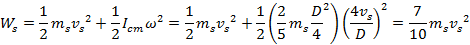


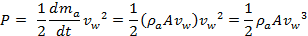


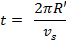


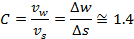


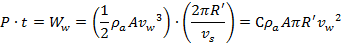


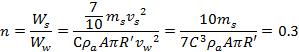


**Section 3.**

**Video S1**. WR-TENG turns on the 30 LEDs in the wind velocity of 22.5 m/s
